# SARS-CoV-2 cases reported from long-term residential facilities (care homes) in South Africa: a retrospective cohort study

**DOI:** 10.1186/s12889-022-13403-6

**Published:** 2022-05-24

**Authors:** Tracy Arendse, Beverley Cowper, Cheryl Cohen, Maureen Masha, Stefano Tempia, Civil Legodu, Sandhya Singh, Tebogo Ratau, Leon Geffen, Ansie Heymans, Dane Coetzer, Lucille Blumberg, Waasila Jassat

**Affiliations:** 1 Division of Public Health Surveillance and Response, National Institute for Communicable Diseases, Johannesburg, South Africa; 2 Right to Care, Johannesburg, South Africa; 3 Sanofi, Johannesburg, South Africa; 4 Centre for Respiratory Diseases and Meningitis, National Institute for Communicable Diseases, Johannesburg, South Africa; 5 School of Public Health, Faculty of Health Sciences, University of the Witwatersrand, Johannesburg, South Africa; 6 National Department of Social Development, Pretoria, South Africa; 7 National Department of Health, Pretoria, South Africa; 8 Life Esidimeni, Johannesburg, South Africa; 9 Samson Institute for Ageing Research, Cape Town, South Africa; 10 Albertina & Walter Sisulu Institute of Ageing in Africa, Geriatrics Unit, Department of Medicine, Faculty of Health Sciences, University of Cape Town, Cape Town, South Africa; 11 South African Council for Social Workers, Pretoria, South Africa; 12 South African Nursing Council, Pretoria, South Africa

**Keywords:** Long term care facilities, Outbreak, Case fatality ratio, SARS-CoV-2, Residents, Staff

## Abstract

**Background:**

Globally, long-term care facilities (LTCFs) experienced a large burden of deaths during the COVID-19 pandemic. The study aimed to describe the temporal trends as well as the characteristics and risk factors for mortality among residents and staff who tested positive for SARS-CoV-2 in selected LTCFs across South Africa.

**Method:**

We analysed data reported to the DATCOV sentinel surveillance system by 45 LTCFs. Outbreaks in LTCFs were defined as large if more than one-third of residents and staff had been infected or there were more than 20 epidemiologically linked cases. Multivariable logistic regression was used to assess risk factors for mortality amongst LTCF residents.

**Results:**

A total of 2324 SARS-CoV-2 cases were reported from 5 March 2020 through 31 July 2021; 1504 (65%) were residents and 820 (35%) staff. Among LTCFs, 6 reported sporadic cases and 39 experienced outbreaks. Of those reporting outbreaks, 10 (26%) reported one and 29 (74%) reported more than one outbreak. There were 48 (66.7%) small outbreaks and 24 (33.3%) large outbreaks reported. There were 30 outbreaks reported in the first wave, 21 in the second wave and 15 in the third wave, with 6 outbreaks reporting between waves. There were 1259 cases during the first COVID-19 wave, 362 during the second wave, and 299 during the current third wave.

The case fatality ratio was 9% (138/1504) among residents and 0.5% (4/820) among staff. On multivariable analysis, factors associated with SARS-CoV-2 mortality among LTCF residents were age 40–59 years, 60–79 years and ≥ 80 years compared to < 40 years and being a resident in a LTCF in Free State or Northern Cape compared to Western Cape. Compared to pre-wave 1, there was a decreased risk of mortality in wave 1, post-wave 1, wave 2, post-wave 2 and wave 3.

**Conclusion:**

The analysis of SARS-CoV-2 cases in sentinel LTCFs in South Africa points to an encouraging trend of decreasing numbers of outbreaks, cases and risk for mortality since the first wave. LTCFs are likely to have learnt from international experience and adopted national protocols, which include improved measures to limit transmission and administer early and appropriate clinical care.

**Supplementary Information:**

The online version contains supplementary material available at 10.1186/s12889-022-13403-6.

## Background

The COVID-19 pandemic has greatly impacted on long-term care facilities (LTCFs), with outbreaks reported in many countries, affecting residents, staff and visitors. In the early period of the pandemic, countries in Europe and North America reported that a significant proportion of the total number of deaths due to the SARS-CoV-2 infection occurred in nursing homes [1, 2]. Once SARS-CoV-2 was introduced into an LTCF, it spread quickly resulting in significant morbidity, hospitalization, and mortality [3]. Close living conditions put this population at risk for SARS-CoV-2 transmission. Advanced age and the presence of comorbid conditions among residents are risk factors for poor COVID-19 outcomes [1, 4].

The LTCF structure in South Africa is complex, fragmented and largely based on care of the elderly. South Africa provides old-age pensions to persons who are financially disadvantaged [5], and all persons aged 60 and older are eligible for free primary healthcare, while access to hospital care is free only for those who are not able to afford it [6, 7]. In South Africa, there are an estimated 1150 public and 1000 private residential LTCFs for older persons [7]. In addition to old age and retirement homes, many people are housed in congregate settings such as mental health and substance abuse facilities. The availability of LTCFs reflects urban-rural and historical racial divides, and most are managed by non-governmental and faith-based organisations. The standard of care in these facilities varies in quality [7].

South Africa experienced a first wave of COVID-19 that peaked in July 2020, a second wave that peaked in January 2021 and a third wave that peaked in July 2021. By 9 September 2021, 2,764,931 SARS-CoV-2 cases and 87,015 deaths had been reported [8].

There is currently very little literature on the impact of COVID-19 in LTCFs in low- and middle-income countries (LMIC). It would be important to understand the nature of outbreaks occurring in LTCFs. The aim of this study was to describe the temporal trends in SARS-CoV-2 cases in LTCFs, as well as the demographics, characteristics and risk factors for mortality among residents and staff who tested positive for SARS-CoV-2 in 45 LTCFs across South Africa.

## Methods

### Study design

We implemented a retrospective cohort analysis of SARS-CoV-2 positive cases in LTCFs across South Africa from 5 March 2020–31 July 2021.

### Data source

DATCOV, a hospital surveillance system for COVID-19 admissions, was initiated on 1 April 2020 and then subsequently expanded to include sentinel surveillance in LTCFs, implemented on 4 June 2020. Data are submitted by LTCFs that have agreed to report COVID-19 cases via the DATCOV LTCFs module. Participation in the LTCFs surveillance was voluntary and included a small number of sentinel facilities. When new facilities enrolled, they captured historical cases going back to their first recorded SARS-CoV-2 case.

### Definitions of LTCFs

A range of LTCFs or congregate settings were included in the sentinel surveillance, including old age homes (21, 46.7%), retirement villages (11, 24.4%), mental health facilities (7, 15.6%), substance abuse recovery facilities (4, 8.9%) and frail care facilities 2 (4.4%). An old age home is defined as a LTCF where residents require daily care in a comfortable, safe and active environment [9]. Retirement villages are defined as accommodating places that provide an independent lifestyle for those who do not need additional living assistance [10]. A frail care centre is defined as a place giving care to those who are unable to care for themselves as a result of a motor-vehicle accident, physical disability, severe stroke or old age [9]. Psychiatric and mental hospitals are defined as specialized hospital-based facilities that provide inpatient care and long-stay residential services for people with mental disorders [9]. These facilities are usually independent and stand-alone, although they may have some links with the rest of the healthcare system. Substance abuse rehabilitation treatment facilities are defined as centres rectifying maladaptive behaviours and providing help with recovery from substance abuse disorders [11].

### Study population

The study population included all LTCF residents and staff in participating LTCFs in South Africa.

For the purpose of this study, we defined outbreaks in a LTCF as follows: [12]

- A sporadic case is defined as a single laboratory confirmed case of SARS-CoV-2 with 14-day period or longer before another laboratory-confirmed case.
- A small outbreak was defined as 2 to 20 confirmed SARS-CoV-2 cases or less than one third of residents or staff of a LTCF infected, within a 14-day period, with an epidemiological link.
- A large outbreak was defined as > 20 confirmed SARS-CoV-2 cases or more than one third of residents or staff of a LTCF infected, within a 14-day period, with an epidemiologic link.

The wave periods were defined by the case incidence data with a national weekly incidence of 30 cases per 100,000 [13] as cut off for start and end of wave periods:

- Pre-wave-1: epidemiologic weeks 10–23 of 2020 (1 March – 6 June 2020)
- First wave: epidemiologic weeks 24–34 of 2020 (7 June – 22 August 2020)
- Post-wave 1: epidemiologic weeks 35–46 of 2020 (23 August – 14 November 2020)
- Second wave: epidemiologic weeks 47 of 2020 – week 5 of 2021 (15 November 2020–06 February 2021)
- Post-wave 2: epidemiologic weeks 6–19 of 2021 (7 February – 8 May 2021)
- Third wave: epidemiologic weeks 20–30 of 2021 (09 May 2021–31 July 2021)

### Data collection and management

Data collection was either through direct entry onto the DATCOV online platform or through importation of electronic data via bulk-upload for LTCFs that did not have a stable internet connection. The dedicated data entry clerk in the LTCF would complete the electronic data form for any person who tested positive for SARS-CoV-2. The case reporting form was adapted from the World Health Organisation (WHO) SARS-CoV-2 case reporting tool and included basic demographic data, pre-existing health conditions and outcomes (died and recovered).

Data imports contained validation checks to identify data errors. Routine checks were performed on all data. Missing and discrepant data were followed up telephonically or by email with the submitting person.

COVID-19 mortality was defined as a death related to SARS-CoV-2 that occurred at the LTCF or while being admitted to hospital, excluding deaths that occurred due to other causes or after recovery. A COVID-19 death is defined for surveillance purposes as a death due to a clinically compatible illness, in a confirmed COVID-19 case, unless there is a clear alternative cause of death that cannot be related to COVID disease (e.g. trauma), with no period of complete recovery from COVID-19 between illness and death [14].

### Data analysis

Descriptive statistics including frequencies and percentages were used for categorical variables, while for continuous variables a median and interquartile range (IQR) were calculated.

A random effect multivariable logistic regression model was used to assess risk factors for mortality amongst LTCF residents with laboratory-confirmed SARS-CoV2. Age, race, sex and comorbidities (hypertension, diabetes mellitus, chronic cardiac disease, asthma, other chronic respiratory disease, chronic renal disease, malignancy in the past 5 yrs, HIV, past and current tuberculosis), smoking, obesity, province, type of LTCF, and wave period, were included in models assessing risk factors for COVID-19 mortality. We assessed all variables that were significant at *p* < 0.2 on univariate analysis and dropped non-significant factors (*p* ≥ 0.05) with manual backward elimination. Pairwise interactions were assessed by inclusion of product terms for all variables remaining in the final multivariable additive model. We also reported the univariate association of all covariates evaluated in the analyses described above to the main outcome (mortality in individuals with COVID-19). The statistical analysis was implemented using Stata 15 (Stata Corp®, College Station, Texas, USA).

### Ethical considerations

The data used for this study were de-identified to ensure confidentiality. All personal information of the residents and staff, concerning health status, treatment or stay in a health establishment, were kept confidential and stored in a secure server. For analysis, patient identifiers were de-linked from other data and stored separately. Ethical approval for this study was obtained from the University of the Witwatersrand Human Research Ethics Committee (M160667). The study was performed in accordance with all relevant ethical guidelines and regulations and with good clinical practice.

Disease surveillance is a critical function of the NICD as a statutory body in South Africa. The NICD has a national mandate to conduct COVID-19 hospital surveillance. The amended regulations that accompany the declaration of a national disaster (Disaster Management Act 2002), provides for healthcare institutions to submit data to NICD on notifiable medical conditions, which includes COVID-19. This encompasses the submission of patient details, their treatment and outcomes. In this case individual consent is therefore waived.

## Results

As of 31 July 2021, 2324 SARS-CoV-2 cases were reported from 45 LTCFs in eight of nine provinces in South Africa. Psychiatric facilities reported the most cases (918, 39.5%), followed by old age homes (420, 18.1%), retirement villages (405, 17.4%), substance abuse recovery facilities (391, 16.8%) and frail care facilities (190, 8.2%).

**Fig. 1:**
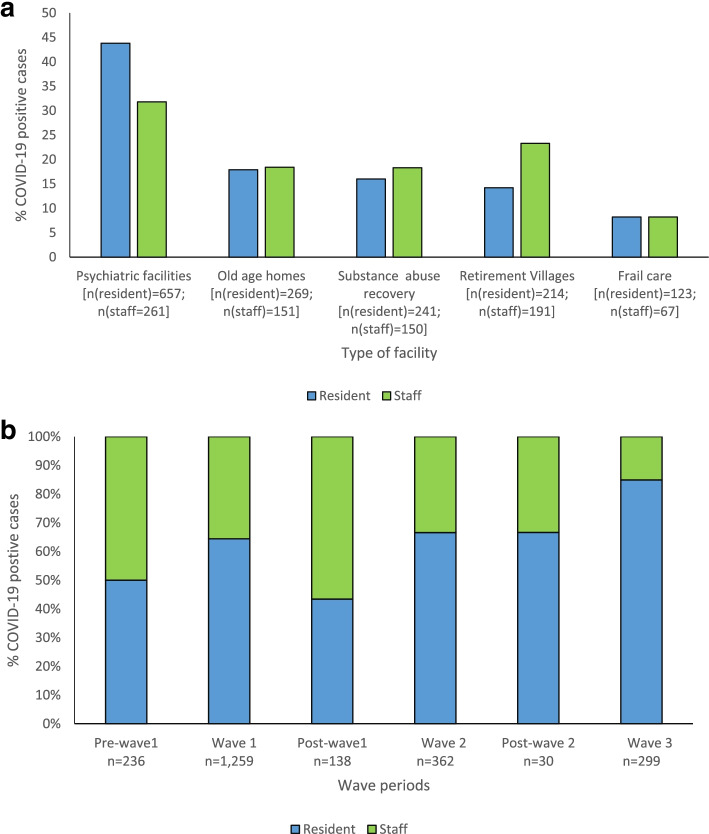
**a.** Proportion of SARS-CoV-2 infected LTCF residents and staff per facility type, South Africa, 5 March 2020–31 July 2021, *n* = 2324. **b.** Proportion of LTCFs resident and staff infected with SARS-CoV-2 5 March 2020–31 July, 2021

Psychiatric facilities reported cases in 657/1504 (43.8%) of residents and 261/820 1504 (31.8%) of staff; old age homes reported 269/1504 (17.9%) residents and 151/820 (18.4%) staff infected, substance abuse recovery centres 241/1504 (16.0%) residents and 150/820 (18.3%) staff infected, retirement villages 214/1504 (14.2%) residents and 191/820 (23.3%) staff infected; and frail care centres 123/1504 (8.2%) residents and 67/820 (8.2%) staff infected (Fig. 1 a).

**Fig. 2:**
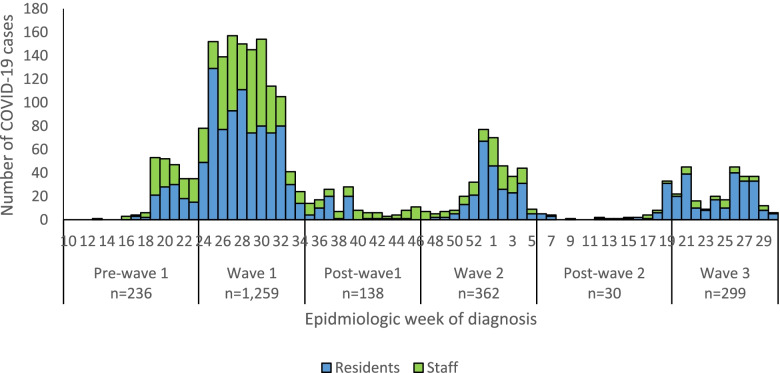
Number of SARS-CoV-2 cases in care homes by epidemiologic week, 5 March 2020–31 July 2021, South Africa, *n* = 2324

Among all SARS-CoV-2 cases from sentinel LTCFs, 1259 (54.2%) were reported during the first wave, 362 (15.6%) during the second wave and 299 (12.9%) during the third wave (Fig. 2).

### Temporal trend in LTCF SARS-CoV-2 cases in LTCFs

The proportion of cases in staff decreased over the surveillance period (Fig. 1 b).

### Pattern of outbreaks in LTCFs

There were varying patterns of outbreaks among LTCFs, with six (13.3%) reporting no outbreaks (only sporadic cases), 10 (22.2%) reporting one outbreak and 29 (64.5%) reporting more than one outbreak (Supplementary Table 1). The six (13.3%) LTCFs reporting sporadic SARS-CoV-2 positive cases included four old age homes and two retirement villages (Supplementary Figure a). There were 48 (66.7%) small outbreaks and 24 (33.3%) large outbreaks reported (Supplementary Figure b). The 24 (61.5%) LTCFs that reported large SARS-CoV-2 outbreaks included 8 (33.3%) retirement villages, 6 (25.0%) psychiatric facilities, 5 (20.8%) old age homes, 4 (16.7%) substance abuse recovery centres and 1 (4.0%) frail care centre (Supplementary Figure c). There were 30 outbreaks reported in wave 1, 21 in wave 2 and 15 in wave 3, while 6 outbreaks were reported between waves.

### Demographic and clinical characteristics of SARS-CoV-2 cases among LTCF residents

The median age of COVID-19 cases among residents was 56 years (IQR 38–73) and 812/1504 (54.0%) were male (Table 1). Of the 1501 (99.8%) residents for whom race was known, 734 (49.0%) were Black African, 610 (40.6%) were White, 101 (6.7%) were Coloured and 56 (3.7%) were Indian. Among 1473 (97.9%) residents for whom there were data on comorbidities, 274 (18.6%) had comorbid conditions. Of these, 191 (69.7%) had one comorbid condition, 60 (21.9%) had two comorbid conditions and 23 (8.3%) had three or more comorbid conditions. The most common comorbid conditions among residents were hypertension (194, 15.2%), diabetes mellitus (64, 4.5%) and chronic cardiac disease (48, 3.4%). Of the 1504 SARS-CoV-2 positive residents with outcomes, 1308 (87.0%) recovered, 59 (3.9%) were active cases and 137 (9.1%) had died, giving a case fatality ratio (CFR) of 9.1%. The CFR excluding active cases amongst residents was 137/1445 (9.5%).

**Table 1:** Characteristics of SARS-CoV-2 cases amongst residents and staff in LTCFs, 5 March 2020–31 July 2021 (*n* = 2324)

Characteristic	Residents ***N*** = 1504 n (%)	Staff ***N*** = 820 n (%)
**Age group**		
0–39 years	400 (26.6)	329 (40.1)
40–59 years	423 (28.1)	444 (54.2)
60–79 years	456 (30.3)	47 (5.7)
80 years	225 (15.0)	0 (0)
**Sex**		
Female	692 (46.0)	705 (86.0)
Male	812 (54.0)	115 (14.0)
**Race**		
White	610 (40.6)	40 (5.2)
Black African	734 (48.9)	640 (83.4)
Mixed	101 (6.7)	79 (10.3)
Indian	56 (3.7)	8 (1.1)
**Comorbidities**		
Hypertension	194 (13.2)	69 (8.7)
Diabetes	64 (4.3)	28 (3.5)
Chronic Cardiac Disease	48 (3.3)	1 (0.1)
Asthma/Chronic Pulmonary Disease	27 (1.8)	14 (1.8)
Chronic Renal Disease	8 (0.5)	0 (0)
Malignancy	9 (0.6)	3 (0.4)
HIV	29 (2.0)	15 (2.0)
Active Tuberculosis	0	1 (0.1)
Past tuberculosis	1 (0.1)	0
**Facility type**		
Substance abuse recovery centre	241 (16.0)	150 (18.3)
Frail care centre	123 (8.2)	67 (8.2)
Old age home	269 (17.9)	151 (18.4)
Psychiatric/Mental	657 (43.7)	261 (31.8)
Retirement village	241 (14.2)	150 (23.3)
**Wave period**		
Pre-wave 1	118 (7.9)	118 (14.4)
Wave 1	811 (53.9)	448 (54.6)
Post-wave 1	60 (4.0)	78 (9.5)
Wave 2	241 (16.0)	121 (14.7)
Post-wave 2	20 (1.3)	10 (1.2)
Wave 3	254 (16.9)	45 (5.5)
**Outcome**		
Recovered	1366 (88.4)	816 (93.0)
Died	138 (9.3)	4 (0.5)

### Demographic and clinical characteristics of SARS-CoV-2 cases among LTCF staff

The median age of COVID-19 admissions amongst staff was 42 years (IQR 35–51) and 705/820 (86.0%) were female (Table 1). Of the 767 (93.5%) staff for whom race was known, 640 (83.4%) were Black African, 79 (10.3%) were Coloured, 8 (1.0%) were Indian and 40 (5.2%) were White. Among 793 (96.7%) staff for whom there were data on comorbidities, 104 (13.1%) had comorbid conditions. Of these, 82 (78.8%) had one comorbid condition, 20 (18.2%) had two and three (2.9%) had three or more comorbid conditions. The most common comorbid conditions among staff were hypertension 69 (8.7%), diabetes mellitus 28 (3.5%), HIV 15 (1.9%) and asthma 14 (1.8%). Of the 820 SARS-CoV-2 positive staff with outcomes, 762 (92.9%) cases recovered, 54 (6.6%) were active cases and 4 (0.5%) had died, giving a case fatality ratio (CFR) of 0.5%. The CFR excluding active cases amongst staff was 4/766 (0.5%).

### Temporal trend in SARS-CoV-2 deaths in LTCFs

**Fig. 3:**
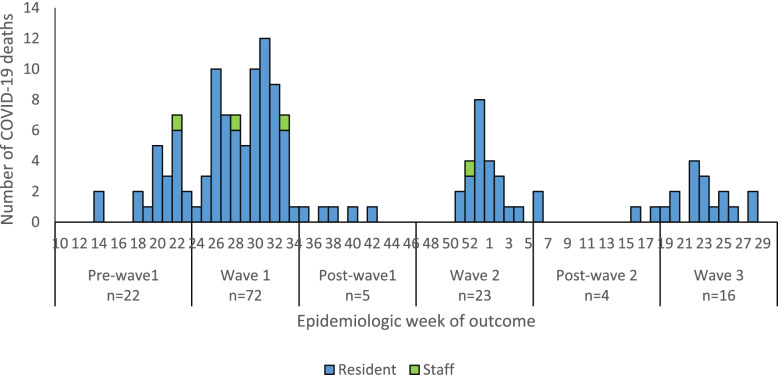
Numbers of COVID-19 deaths reported per week among residents and staff, by epidemiologic week, South Africa, 5 March 2020–31 July 2021, (*n* = 142)

The number of SARS-CoV-2 deaths and CFR among residents in the various epidemic periods were 22/115 (19.1%) in pre-wave 1; 72/793 (9.1%) during wave 1; 5/60 (8.3%) during post-wave 1; 23/241 (9.5%) during wave 2, 4/18 (22.2%) during post-wave 2 and 16/218 (7.3%) during wave 3. (Fig. 3).

The number of SARS-CoV-2 deaths and CFR among staff in the various pandemic periods were 1/91 (1.1%) during pre-wave 1; 2/438 (0.5%) during wave 1; and 1/112 (0.9%). No deaths occured in satff during post-wave 1, post-wave 2 and wave 3.

Among residents the CFR was 46/252 (18.3%) in old age homes, 15/112 (13.4%) in frail care centres; 21/389 (10.5%) in retirement villages; 47/649 (7.2%) in psychiatric facilities; and 9/232 (3.9%) in substance abuse recovery centres.

Of the four staff (0.5%) who died, two each were female and male. The median age was 48.5 years (IQR 44.5–57.0). One female had hypertension and diabetes mellitus. The remaining three individuals who died did not have any reported comorbidities. Each of the four deaths occurred in an old age home, psychiatric facility, retirement village and a substance abuse recovery centre.

### Factors associated with SARS-CoV-2 mortality in residents

On multivariable analysis, factors associated with SARS-CoV-2 mortality among LTCF residents were age 40–59 years (aOR 2.4, 95% CI 1.0–5.5), 60–79 years (aOR 7.6, 95% CI 3.6–16.5) and ≥ 80 years (aOR 18.2, 95% CI 8.1–41.2) compared to < 40 years; and being a resident in a LTCF in Free State (aOR 4.2, 95% CI 1.4–12.8) or Northern Cape (aOR 3.8, 95% CI 1.2–12.6) compared to Western Cape (Table 2). Compared to pre-wave 1, there was a decreased risk of mortality in wave 1 (aOR 0.3, 95% CI 0.1–0.9), post-wave 1 (aOR 0.2, 95% CI 0.05–0.8), wave 2 (aOR 0.3, 95% CI 0.1–1.0), post-wave 2 (aOR 0.1, 95% CI 0.02–0.9) and wave 3 (aOR 0.2, 95% CI 0.006–0.6).

**Table 2:** Multivariable analysis of factors associated with SARS-CoV-2 mortality among LTCF residents, South Africa, 5 March 2020–31 July 2021 (*n* = 1504)

Characteristic	Case fatality ratio n/N (%)	Unadjusted odds ratio (95% CI)	p-value	Adjusted odds ratio (95% CI)	***p***-value
**Age group**					
0-39 years	8/400 (2.0)	Reference		Reference	
40-59 years	20/423 (4.7)	2.4 (1.1-5.6)	0.036	2.4 (1.0-5.5)	0.038
60-79 years	58/456 (12.7)	7.1 (3.4-15.2)	≤0.001	7.6 (3.6-16.5)	≤0.001
≥ 80 years	52/225 (23.1)	14.7 (6.8-31.7)	≤0.001	18.2 (8.1-41.2)	≤0.001
**Sexs**					
Female	59/692 (8.7)	Reference			
Male	78/812 (9.9)	1.1 (0.8-1.6)	0.531		
**Race/Ethnicity**					
Black	47/734 (6.4)	Reference			
Coloured	8/101 (7.9)	1.3 (0.6-2.8)	0.572		
White	74/610 (12.1)	2.8 (1.3-6.2)	0.008		
Indian	9/56 (16.1)	2.1 (1.4-3.0)	≤0.001		
**Type of LTCF**					
Substance Abuse	9/241 (3.7)	Reference			
Frail Care Centre	16/123 (13.0)	3.9 (1.7-9.0)	0.002		
Old age home	45/269 (16.7)	5.1 (2.5-11.0)	≤0.001		
Psychiatric/Mental	47/657 (7.2)	2.0 (0.9-4.1)	0.065		
Retirement village	21/214 (9.8)	2.8 (1.3-6.3)	0.012		
**Wave period**					
Pre-wave 1	21/118 (18.3)	Reference		Reference	
Wave 1	70/811 (8.6)	0.4 (0.3-0.7)	0.002	0.3 (0.1-0.9)	0.023
Post-wave 1	5/60 (8.3)	0.4 (0.1-1.1)	0.099	0.2 (0.05-0.8)	0.027
Wave 2	25/241 (10.4)	0.5 (0.3-1.0)	0.051	0.3 (0.1-1.0)	0.059
Post-wave 2	2/20 (10.0)	0.5 (0.1-2.4)	0.394	0.1 (0.02-0.9)	0.035
Wave 3	15/254 (5.9)	0.3 (0.1-0.6)	0.001	0.2 (0.06-0.6)	0.004
**Comorbid condition**					
1 comorbid condition	22/191 (11.5)	Reference			
2 comorbid conditions ≥ 3 comorbid conditions	10/60 (16.7) 5/24 (20.8)	1.5 (0.7-3.5) 2.0 (0.7-6.0)	0.300 0.202		
**Hypertension**					
No	108/1,280 (8.4)	Reference			
Yes	27/194 (13.9)	1.8 (1.1-2.8)	0.015		
**Diabetes mellitus**					
No	123/1,410 (8.7)	Reference			
Yes	12/64 (18.8)	2.4 (1.3-4.6)	0.008		
**Chronic cardiac disease**					
No	132/1,466 (9.0)	Reference			
Yes	3/8 (37.5)	2.7 (1.3-5.6)	0.006		
**Asthma/Chronic pulmonary disease**					
No	134/1,450 (9.2)	Reference			
Yes	1/24 (4.2)	0.4 (0.1-3.1)	0.407		
**Chronic renal disease**					
No	132/1,466 (9.0)	Reference			
Yes	3/8 (37.5)	6.1 (1.4-25.74)	0.014		
**Malignancy**					
No	134/1,465 (9.2)	Reference			
Yes	1/9 (11.1)	1.2 (0.2-10.0)	0.839		
**HIV**					
No	133/1,445 (9.2)	Reference			
Yes	2/29 (6.9)	0.7 (0.2-3.1)	0.671		
**Active Tuberculosis**					
No	135/1,474 (9.2)	Reference			
Yes	0	1	─		
**Past Tuberculosis**					
No	135/1,473 (9.2)	Reference			
Yes	0	1	─		
**Smoking**					
Never smoked	38/412 (9.2)	Reference			
Former smoker	8/70 (11.4)	1.3 (0.6-2.9)	0.461		
Current smoker	2/209 (1.0)	0.09 (0.02-0.4)	0.001		
**Province**					
Western Cape	14/64 (21.8)	Reference		Reference	
Eastern Cape	12/132 (9.1)	0.4 (0.2-0.8)	0.016	1.3 (0.5-3.1)	0.620
Free State	30/225 (13.3)	0.5 (0.3-1.1)	0.097	4.2 (1.4-12.8)	0.012
Gauteng	26/430 (6.1)	0.2 (0.1-0.5)	≤0.001	1.5 (0.5-4.7)	0.441
KwaZulu-Natal	27/305 (8.9)	0.3 (0.2-0.7)	0.004	1.6 (0.5-5.0)	0.380
Limpopo	2/35 (5.7)	0.2 (0.04-1.1)	0.052	1.5 (0.2-9.6)	0.655
Mpumalanga	10/99 (10.1)	0.4 (0.2-0.9)	0.043	1.5 (0.4-4.8)	0.544
Northern Cape	17/214 (7.9)	0.3 (0.1-0.7)	0.003	3.8 (1.2-12.6)	0.028

Table 2 Multivariable analysis of factors associated with SARS-CoV-2 mortality among LTCF residents, South Africa, 5 March 2020–31 July 2021 (*n* = 1504).

## Discussion

The DATCOV sentinel surveillance system has provided data on the pattern of SARS-CoV-2 outbreaks among 45 LTCFs in South Africa. From April 2020 to July 2021, 15 LTCFs reported one outbreak and 24 reported two or more outbreaks; of those that reported an outbreak, 10 (25.6%) reported small outbreaks and 29 (64.4%) reported large outbreaks. This analysis has revealed a decreasing trend in the numbers of SARS-CoV-2 outbreaks and cases in LTCFs over the course of the epidemic, with the highest risk of mortality occurring in the very early weeks of the epidemic.

Outbreaks were more frequently reported during the first COVID-19 wave, and the numbers of cases among LTCFs were lower in the second and third waves. This is notable given that the second wave in South Africa was associated with more cases and deaths and higher in-hospital CFR than the first wave [15]. Other countries have similarly reported fewer cases, deaths and CFR in LTCFs in the second wave [12, 16]. This is ascribed to improved control measures and shielding of vulnerable people. Firstly, there was greater awareness of fatality risks among the vulnerable LTCFs residents. Secondly, efforts were put in place to protect LTCFs as learned from experience during the first wave of COVID-19 in nursing homes. Thirdly, improved routine hygiene measures, infection control, testing of personnel and residents, and avoidance of staff working across multiple nursing homes were implemented during the second wave [17]. Another reason for lower number of deaths during the second and third waves, may be due to most at risk, frail people having died during the first wave.

The CFR amongst residents with highest risk in old age homes (18%) and frail care centres (13%), was lower than rates reported in other studies [18]. The residents in these settings were likely older and had comorbidities which are known risk factors for COVID-19 mortality. A review of COVID-19 in LTCFs reported an average mortality rate of 21% [2]. A population-based analysis of LTCFs in the United Kingdom, reported a case fatality ratio of 48% [17]. The pooled mortality rate in the first three months of the pandemic amongst LTCFs in Spain was 28% [19], In a cohort study of SARS-CoV-2 cases in LTCFs in the UK, 10% of residents and 5% of staff were infected with a CFR in residents of 36% [20]. Another study in the US involving 30 LTCFs, reported a CFR among residents of 34% [21]. These studies reported high CFR in the first few months of the pandemic; our average CFR estimate was lower for the study period over 17 months.

One of the risk factors for mortality among LTCFs residents was age over 40 years. Older age, male sex and the presence of comorbidities such as hypertension, diabetes, chronic cardiac and renal diseases, malignancy, HIV and TB, as well as obesity, have been described as risk factors for COVID-19 mortality in meta-analyses and from a large national surveillance system in South Africa [10, 22, 23].

There highest risk of mortality amongst LTCFs in the early weeks of the epidemic. One contributing factor may be improved preparedness, treatment and case management over time [24]. LTCFs did not have increased mortality in the second and third waves predominated by the Beta and Delta variants respectively. This is in contrast to the increased in-hospital mortality reported in the second wave compared to the first wave in South Africa possibly related to the circulation of the Beta variant which predominated during the second wave [15].

### Strengths and limitations

We could not find any other studies in Africa describing SARS-CoV-2 in LTCFs. DATCOV is a sentinel surveillance system that has only a small number of reporting sites for LTCFs which may not be generalizable across the country, but it does include sites in most provinces and across different LTCF types. There is some incomplete data in the surveillance system, particularly that of smoking (1348/2324, 58.0%). These variables have therefore been excluded from the multivariable analysis.

## Conclusion

While studies from developed countries have reported that infection control interventions, such as universal testing of staff and residents, were effective in mitigating COVID-19 outbreaks [3], these strategies are unlikely to be feasible in LMIC settings. The analysis in sentinel LTCFs in South Africa, the only study of its kind to our knowledge in Africa, points to an encouraging trend of decreasing number of SARS-CoV-2 outbreaks, cases and CFR from the first to the third waves. LTCFs are likely to have learnt from international experience and adopted national protocols, which include improved measures to limit transmission and administer appropriate clinical care.

## Supplementary Information

**Additional file 1.**

## Data Availability

The datasets generated and/or analysed during the current study are not publicly available due to patient/participant confidentiality but are available from the corresponding author on reasonable request. Data are securely stored on the DATCOV surveillance system and is available upon request.

## Abbreviation

CFR: Case Fatality Ratio
COVID-19: Coronavirus Disease 2019
HIV: Human Immunodeficiency Virus
IQR: Inter Quartile Range
LMIC: Low- and Middle-Income Countries
LTCF: Long-term Care Facility
NICD: National Institute for Communicable Diseases
RT-PCR: Real Time Polymerase Chain Reaction
SARS-CoV-2: Severe Acute Respiratory Syndrome Coronavirus Two
TB: Tuberculosis
WHO: World Health Organization
