# Supplementary material for: SARS-CoV-2 cases reported from long-term residential facilities (care homes) in South Africa: a retrospective cohort study
Source: BMC Public Health. 2022 May 24;22:1035. doi: 10.1186/s12889-022-13403-6 (PMC9126632; doi:10.1186/s12889-022-13403-6)
Supplement: Supplementary file 1 — Additional file 1. [file 12889_2022_13403_MOESM1_ESM.pdf]

**Supplementary Table 1.** Number of residents and staff who tested positive for SARS-CoV-2 in 45 LTCFs across South Africa, 5 March 2020 – 12 March 2021, n=2,324

| | Resident | | | Staff | | |
|---|---|---|---|---|---|---|
| | Cases n | Total residents | % infected | Cases n | Total staff | % infected |
| Facility | | | | | | |
| WCRV2 | 8 | 16 | 50.0 | 7 | 25 | 92.6 |
| ECRV4 | 4 | 45 | 8.8 | 11 | 36 | 30.6 |
| MPRV4 | 19 | 267 | 7.1 | 14 | 19 | 73.6 |
| NWOAH1 | 0 | 29 | 0 | 1 | 33 | 3.0 |
| KZNPSYCH1 | 103 | 580 | 22.0 | 0 | 339 | 0 |
| MPRV2 | 1 | 24 | 4.2 | 0 | 0 | 0 |
| FSPSYCHMEN | 155 | 685 | 22.6 | 165 | 903 | 18.3 |
| WCOAH1 | 34 | 157 | 21.7 | 55 | 250 | 22 |
| FSOAH1 | 2 | 50 | 4.0 | 0 | 31 | 0 |
| MPOAH | 2 | 46 | 4.3 | 0 | 43 | 0 |
| MPRV3 | 23 | 67 | 34.3 | 0 | 63 | 0 |
| WCRV1 | 4 | 202 | 2.0 | 25 | 54 | 86.2 |
| GPRV | 51 | 146 | 34.9 | 12 | 131 | 9.2 |
| ECRV1 | 22 | 215 | 10.2 | 35 | 184 | 19.0 |
| ECRV2 | 45 | 465 | 9.6 | 64 | 294 | 21.8 |
| ECPSYCH | 71 | 648 | 10.9 | 44 | 224 | 19.6 |
| GPSARC3 | 39 | 350 | 11.1 | 26 | 155 | 16.7 |
| LPPSYCHMEN | 35 | 160 | 21.9 | 34 | 154 | 22.1 |
| MPSARC | 30 | 250 | 12.0 | 33 | 143 | 23.1 |
| GPSARC1 | 136 | 510 | 26.7 | 66 | 233 | 28.3 |
| ECRV3 | 35 | 120 | 29.2 | 23 | 100 | 23.0 |
| GPSARC2 | 36 | 220 | 16.3 | 25 | 106 | 23.6 |
| MPRV | 2 | 57 | 3.6 | 0 | 20 | 0 |
| GPFCC | 123 | 269 | 45.7 | 65 | 234 | 27.8 |
| FSOAH2 | 41 | 78 | 52.5 | 13 | 31 | 41.9 |
| MPOAH1 | 1 | 58 | 1.7 | 14 | 70 | 20 |
| MPFCC1 | 0 | 46 | 0 | 2 | 43 | 4.7 |
| MPOAH2 | 1 | 105 | 1 | 9 | 49 | 18.4 |
| WCOAH2 | 18 | 27 | 66.7 | 11 | 21 | 53.4 |
| MPOAH10 | 19 | 50 | 38 | 1 | 42 | 2.4 |
| KZNOAH1 | 37 | 609 | 6.0 | 30 | 43 | 69.8 |
| KZNOAH2 | 5 | 77 | 6.5 | 0 | 7 | 0 |
| KZNOAH4 | 11 | 70 | 15.7 | 1 | 51 | 2.0 |
| KZNOAH5 | 11 | 172 | 6.4 | 5 | 57 | 8.8 |
| KZNOAH6 | 6 | 217 | 2.8 | 0 | 11 | 0 |
| KZNOAH7 | 12 | 185 | 6.5 | 2 | 22 | 9.1 |
| KZNOAH8 | 42 | 140 | 30.0 | 5 | 152 | 3.2 |
| KZNPSYCHMEN | 78 | 304 | 25.7 | 18 | 343 | 4.7 |

| | | | | | | |
|---|---|---|---|---|---|---|
| MPOAH9 | 0 | 36 | 0 | 1 | 32 | 3.1 |
| NWPSYCHMEN | 213 | 573 | 42.1 | 0 | 1059 | 0 |
| WCOAH3 | 1 | 80 | 1.3 | 0 | 48 | 2.1 |
| FSOAH3 | 2 | 86 | 2.3 | 0 | 30 | 0 |
| FSOAH4 | 20 | 118 | 16.9 | 0 | 34 | 41.2 |
| FSOAH5 | 5 | 17 | 29.4 | 1 | 14 | 7.1 |
| MPPSYCHMEN1 | 1 | 68 | 1.5 | 2 | 54 | 3.7 |
| Total | 1504 | 8694 | 17.2 | 820 | 5987 | 13.7 |

**Multi-panel figures**

**Sporadic cases among long-term care facilities**

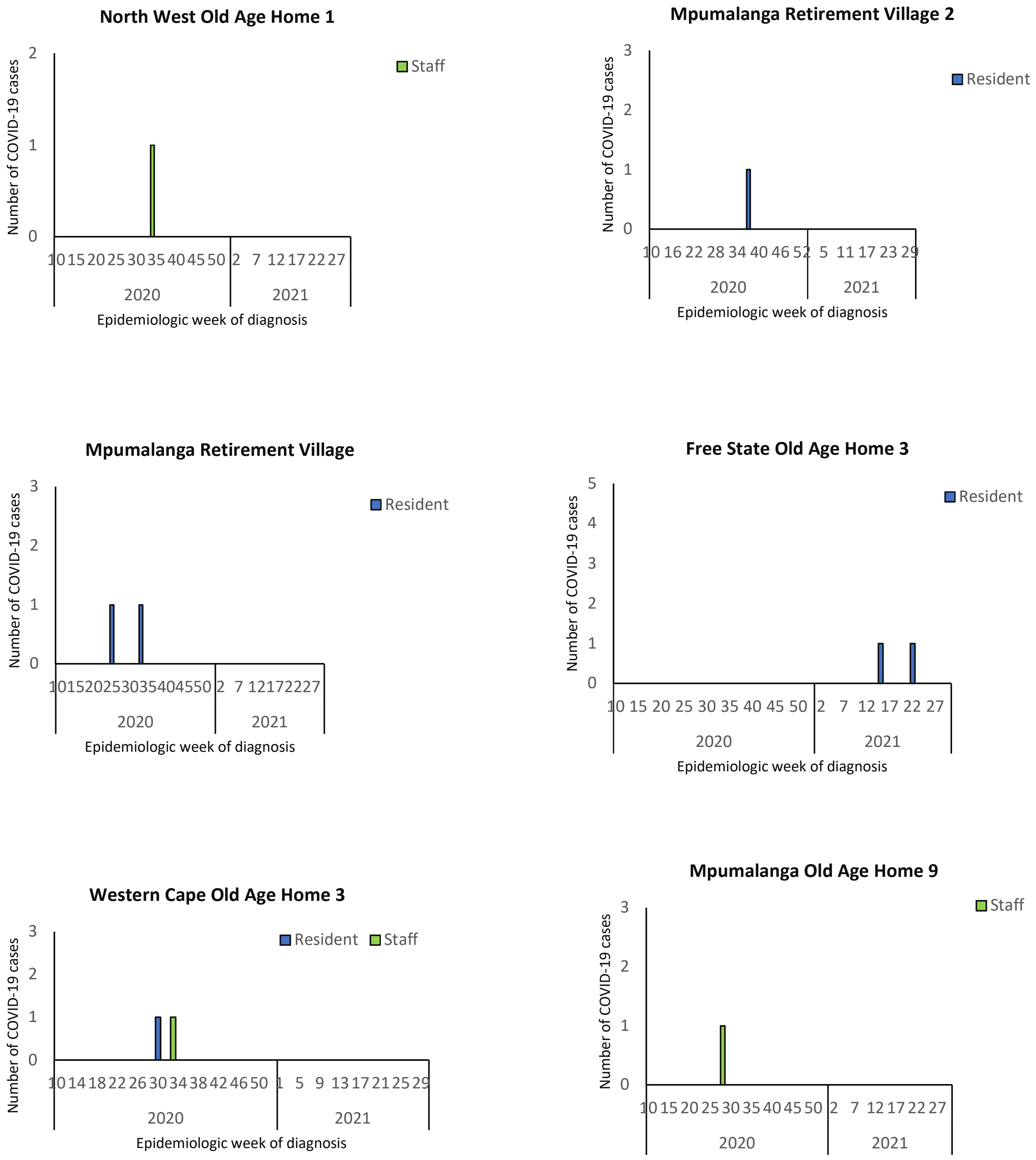


**Supplementary Figure a.** Number of SARS-CoV-2 cases among care homes reporting sporadic SARS-CoV-2 cases, South Africa, 5 March 2020 – 31 July 2021, n=6

## Small outbreaks among long term care facilities

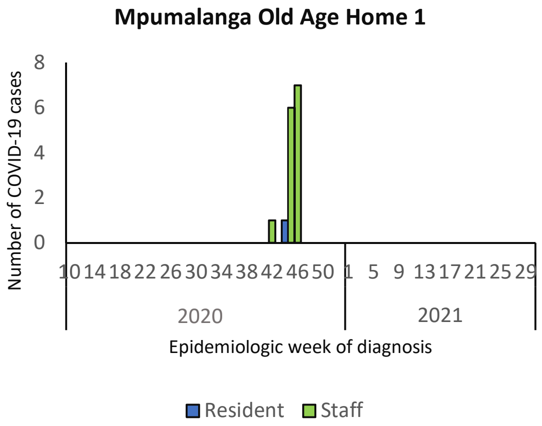


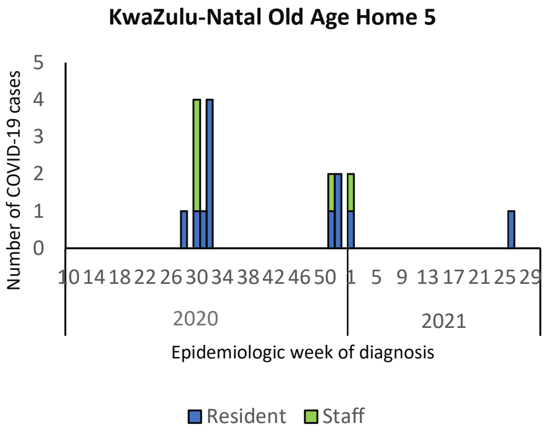


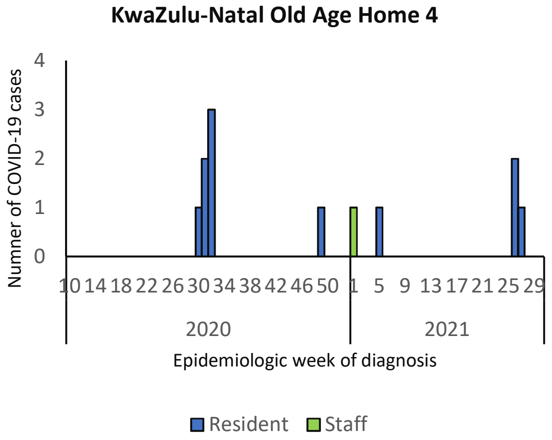


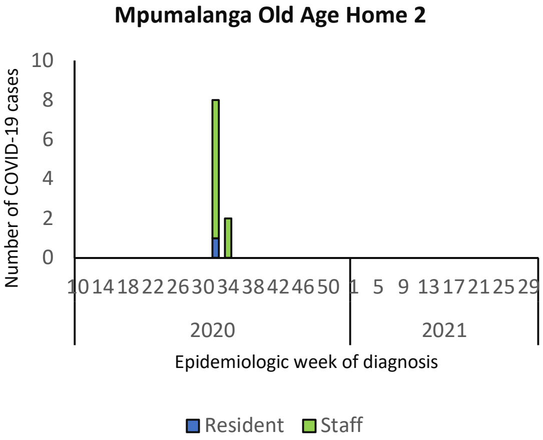


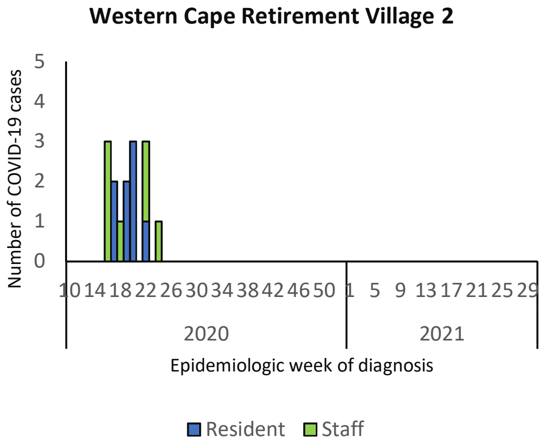


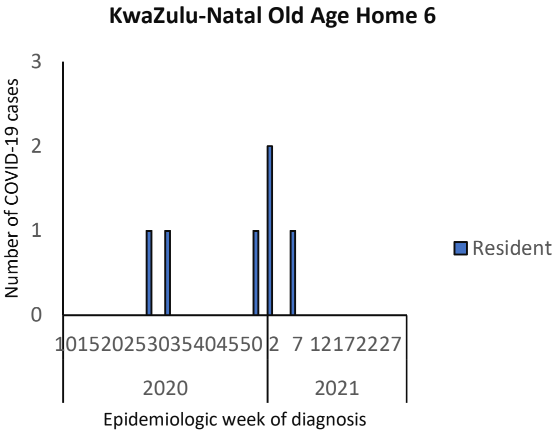

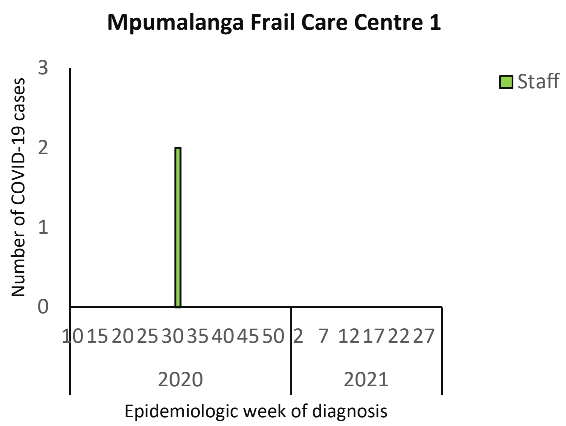
Mpumalanga Frail Care Centre 1
Staff
Number of COVID-19 cases
3
2
1
0
10 15 20 25 30 35 40 45 50 2 7 12 17 22 27
2020
2021
Epidemiologic week of diagnosis

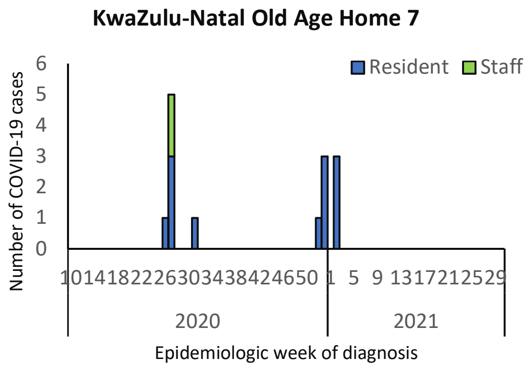
KwaZulu-Natal Old Age Home 7
Resident
Staff
Number of COVID-19 cases
6
5
4
3
2
1
0
10 14 18 22 26 30 34 38 42 46 50 1 5 9 13 17 21 25 29
2020
2021
Epidemiologic week of diagnosis

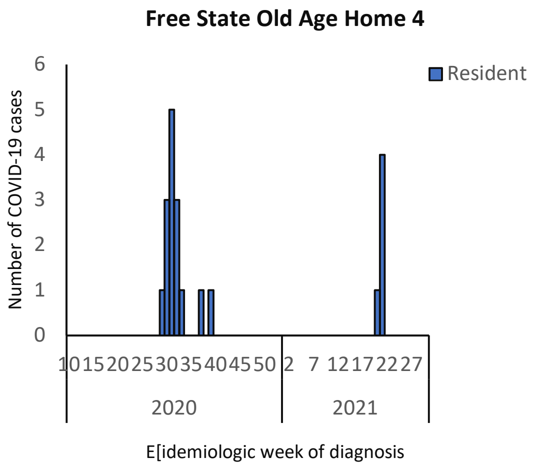
Free State Old Age Home 4
Resident
Number of COVID-19 cases
6
5
4
3
2
1
0
10 15 20 25 30 35 40 45 50 2 7 12 17 22 27
2020
2021
E[idemiologic week of diagnosis

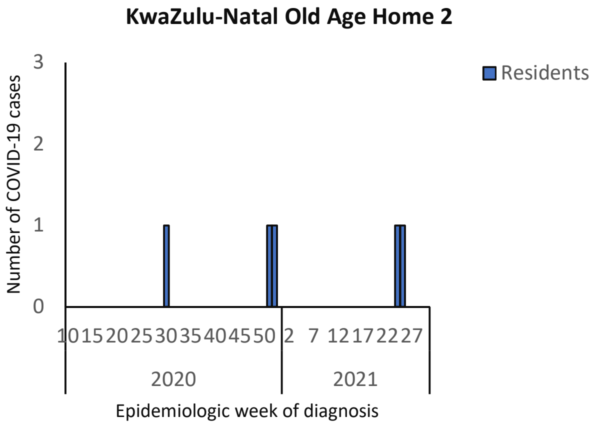
KwaZulu-Natal Old Age Home 2
Residents
Number of COVID-19 cases
3
2
1
0
10 15 20 25 30 35 40 45 50 2 7 12 17 22 27
2020
2021
Epidemiologic week of diagnosis

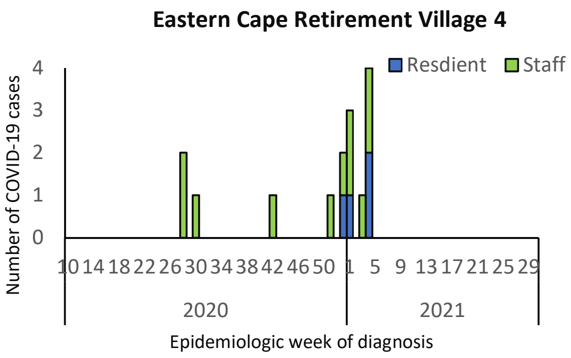
Eastern Cape Retirement Village 4
Resdient
Staff
Number of COVID-19 cases
4
3
2
1
0
10 14 18 22 26 30 34 38 42 46 50 1 5 9 13 17 21 25 29
2020
2021
Epidemiologic week of diagnosis

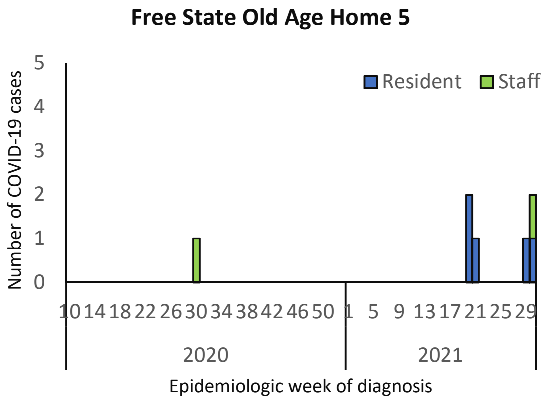
Free State Old Age Home 5
Resident
Staff
Number of COVID-19 cases
5
4
3
2
1
0
10 14 18 22 26 30 34 38 42 46 50 1 5 9 13 17 21 25 29
2020
2021
Epidemiologic week of diagnosis

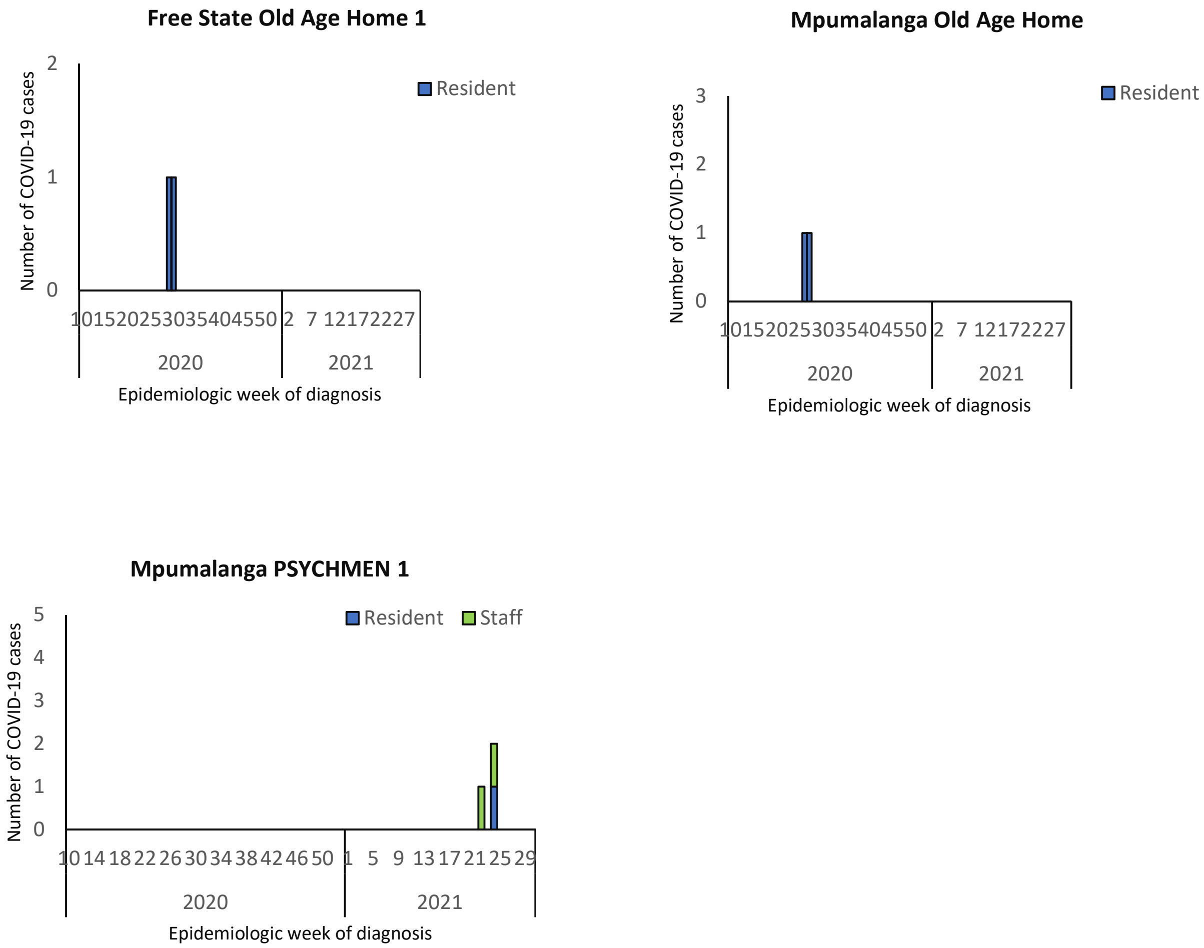


**Supplementary Figure b.** Number of SARS-CoV-2 cases among LTCFs reporting small outbreaks of SARS-CoV-2 cases, South Africa, 5 March 2020 – 31 July 2021, n=15

## Large outbreaks among long-term care facilities

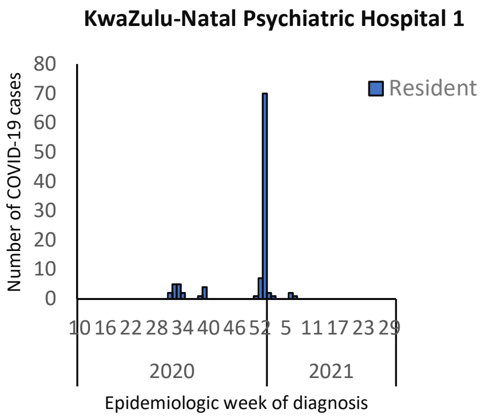


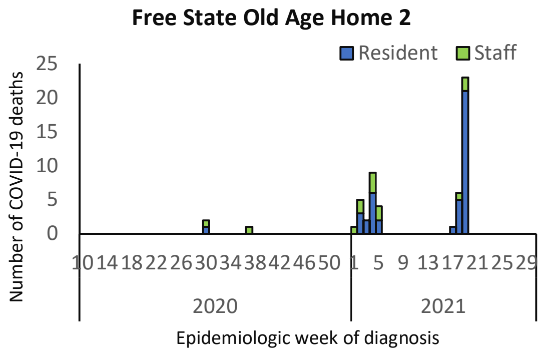


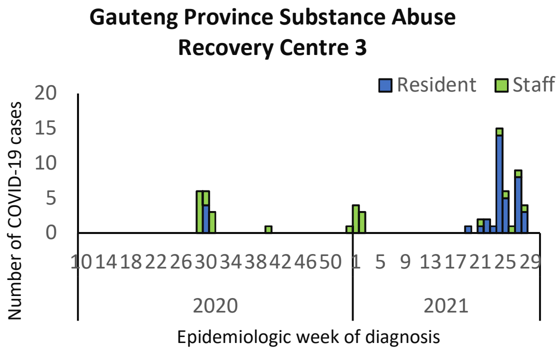


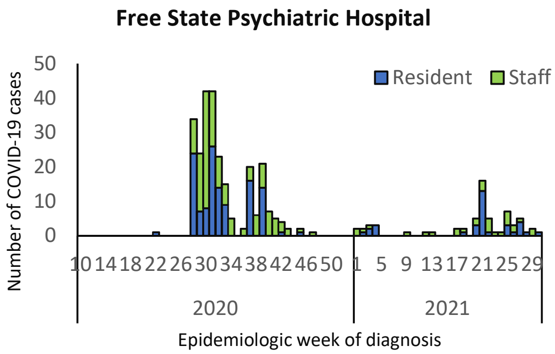


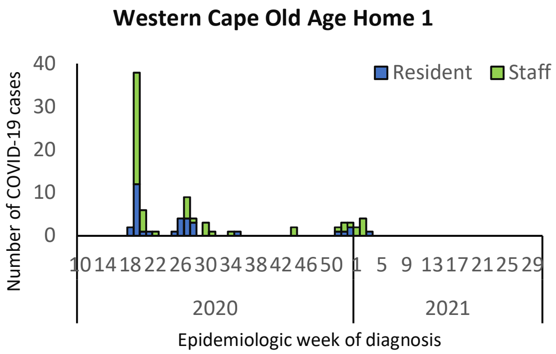


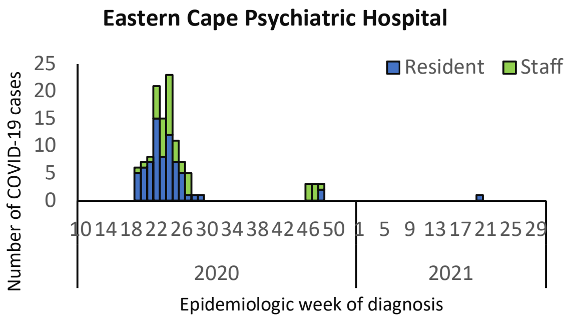

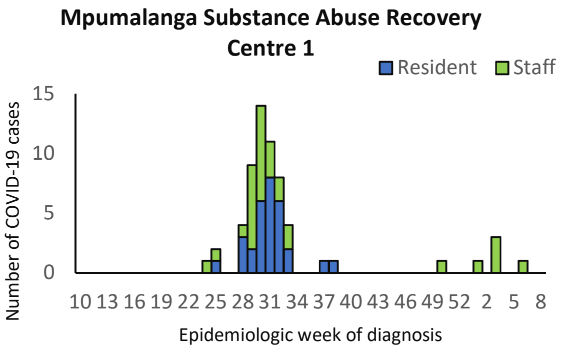
Mpumalanga Substance Abuse Recovery Centre 1
Resident
Staff
Number of COVID-19 cases
0
5
10
15
10 13 16 19 22 25 28 31 34 37 40 43 46 49 52 2 5 8
Epidemiologic week of diagnosis

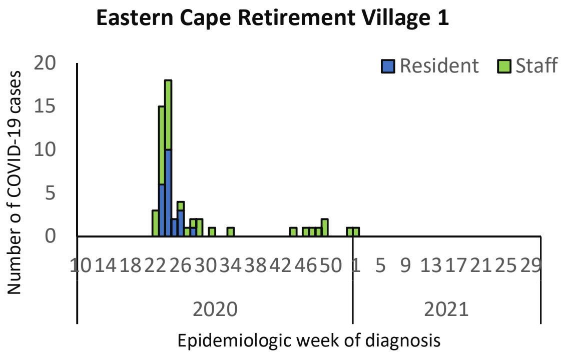
Eastern Cape Retirement Village 1
Resident
Staff
Number o f COVID-19 cases
0
5
10
15
20
10 14 18 22 26 30 34 38 42 46 50 1 5 9 13 17 21 25 29
2020
2021
Epidemiologic week of diagnosis

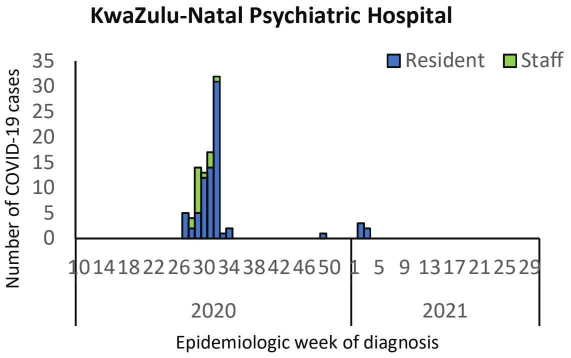
KwaZulu-Natal Psychiatric Hospital
Resident
Staff
Number of COVID-19 cases
0
5
10
15
20
25
30
35
10 14 18 22 26 30 34 38 42 46 50 1 5 9 13 17 21 25 29
2020
2021
Epidemiologic week of diagnosis

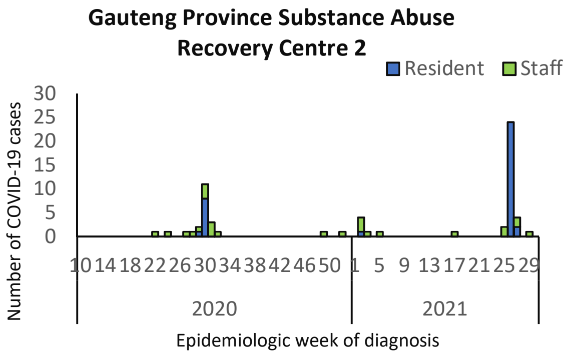
Gauteng Province Substance Abuse Recovery Centre 2
Resident
Staff
Number of COVID-19 cases
0
5
10
15
20
25
30
10 14 18 22 26 30 34 38 42 46 50 1 5 9 13 17 21 25 29
2020
2021
Epidemiologic week of diagnosis

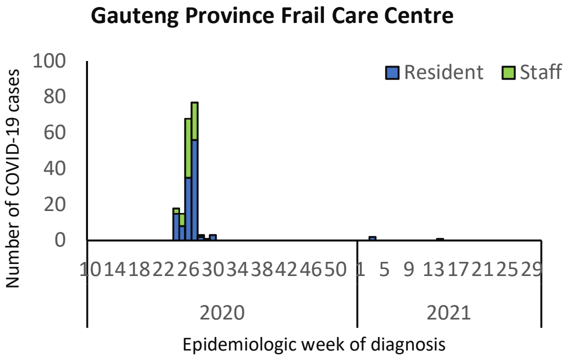
Gauteng Province Frail Care Centre
Resident
Staff
Number of COVID-19 cases
0
20
40
60
80
100
10 14 18 22 26 30 34 38 42 46 50 1 5 9 13 17 21 25 29
2020
2021
Epidemiologic week of diagnosis

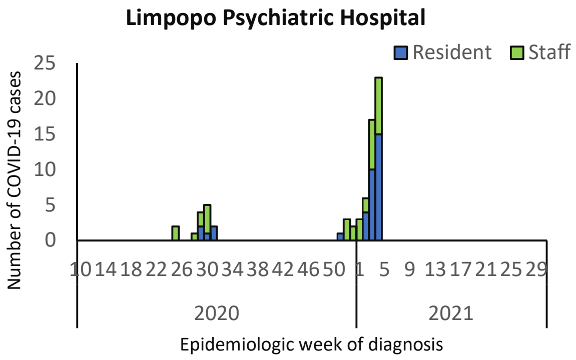
Limpopo Psychiatric Hospital
Resident
Staff
Number of COVID-19 cases
0
5
10
15
20
25
10 14 18 22 26 30 34 38 42 46 50 1 5 9 13 17 21 25 29
2020
2021
Epidemiologic week of diagnosis

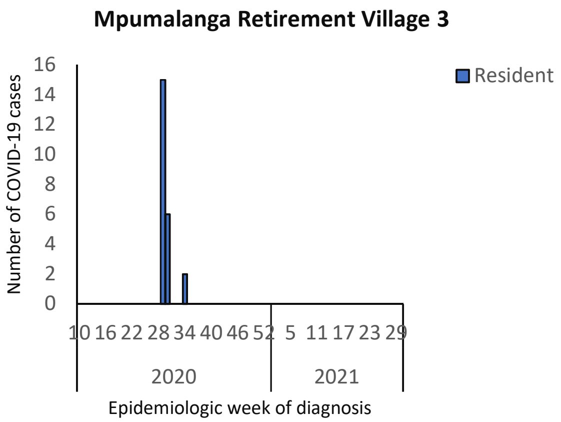
Mpumalanga Retirement Village 3
Resident
Number of COVID-19 cases
16
14
12
10
8
6
4
2
0
10 16 22 28 34 40 46 52 5 11 17 23 29
2020
2021
Epidemiologic week of diagnosis

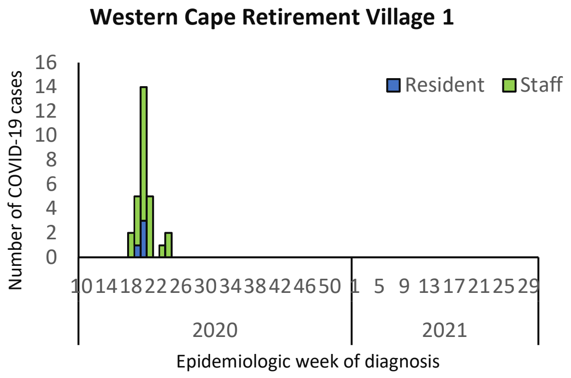
Western Cape Retirement Village 1
Resident
Staff
Number of COVID-19 cases
16
14
12
10
8
6
4
2
0
10 14 18 22 26 30 34 38 42 46 50 1 5 9 13 17 21 25 29
2020
2021
Epidemiologic week of diagnosis

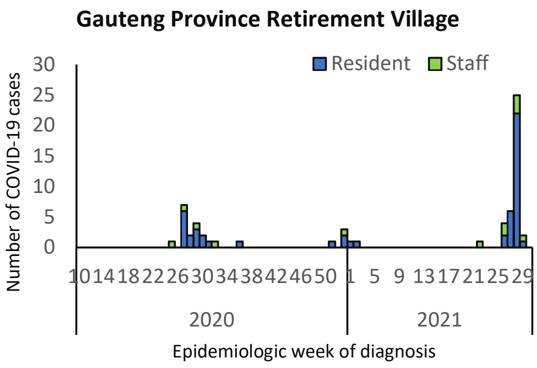
Gauteng Province Retirement Village
Resident
Staff
Number of COVID-19 cases
30
25
20
15
10
5
0
10 14 18 22 26 30 34 38 42 46 50 1 5 9 13 17 21 25 29
2020
2021
Epidemiologic week of diagnosis

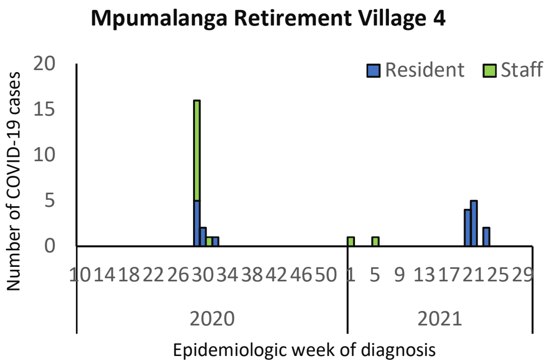
Mpumalanga Retirement Village 4
Resident
Staff
Number of COVID-19 cases
20
15
10
5
0
10 14 18 22 26 30 34 38 42 46 50 1 5 9 13 17 21 25 29
2020
2021
Epidemiologic week of diagnosis

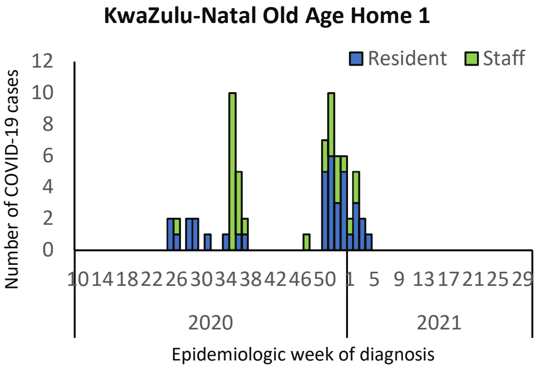
KwaZulu-Natal Old Age Home 1
Resident
Staff
Number of COVID-19 cases
12
10
8
6
4
2
0
10 14 18 22 26 30 34 38 42 46 50 1 5 9 13 17 21 25 29
2020
2021
Epidemiologic week of diagnosis

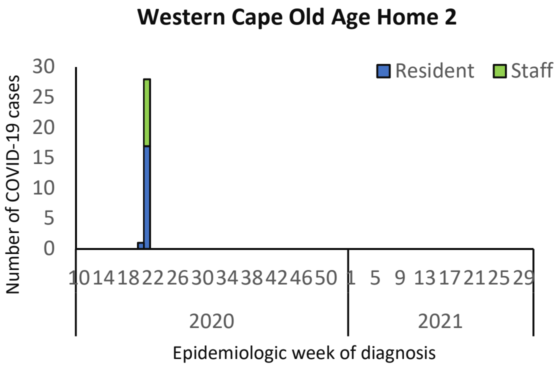
Western Cape Old Age Home 2
Resident
Staff
Number of COVID-19 cases
30
25
20
15
10
5
0
10 14 18 22 26 30 34 38 42 46 50 1 5 9 13 17 21 25 29
2020
2021
Epidemiologic week of diagnosis

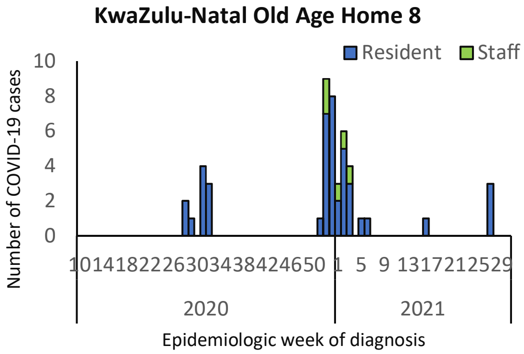
KwaZulu-Natal Old Age Home 8
Resident
Staff
Number of COVID-19 cases
0
2
4
6
8
10
10141822263034384246501 5 9 1317212529
2020
2021
Epidemiologic week of diagnosis

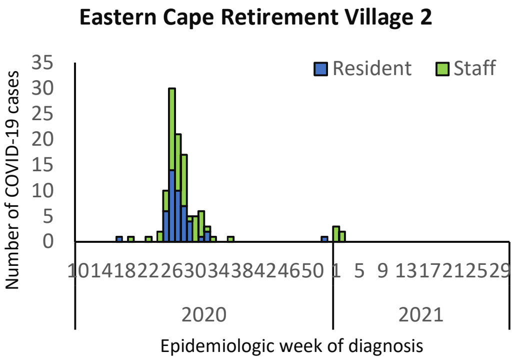
Eastern Cape Retirement Village 2
Resident
Staff
Number of COVID-19 cases
0
5
10
15
20
25
30
35
10141822263034384246501 5 9 1317212529
2020
2021
Epidemiologic week of diagnosis

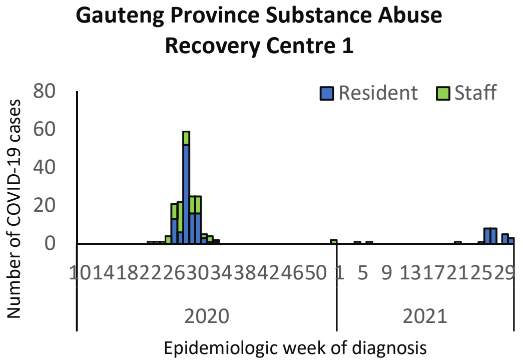
Gauteng Province Substance Abuse Recovery Centre 1
Resident
Staff
Number of COVID-19 cases
0
20
40
60
80
10141822263034384246501 5 9 1317212529
2020
2021
Epidemiologic week of diagnosis

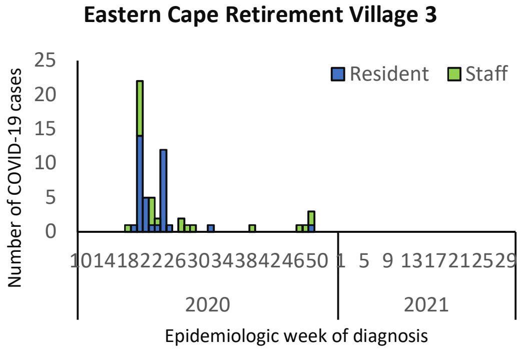
Eastern Cape Retirement Village 3
Resident
Staff
Number of COVID-19 cases
0
5
10
15
20
25
10141822263034384246501 5 9 1317212529
2020
2021
Epidemiologic week of diagnosis

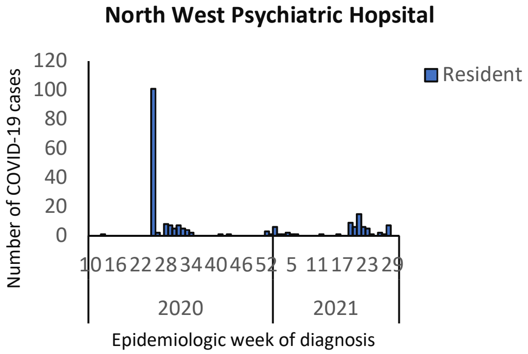
North West Psychiatric Hopsital
Resident
Number of COVID-19 cases
0
20
40
60
80
100
120
10 16 22 28 34 40 46 52 5 11 17 23 29
2020
2021
Epidemiologic week of diagnosis

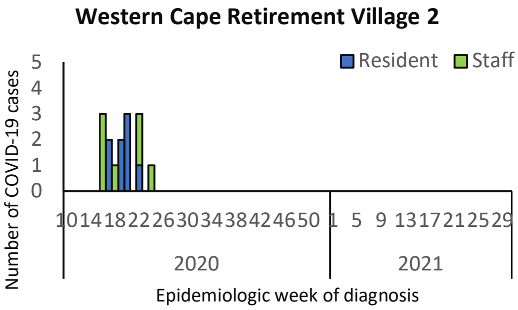
Western Cape Retirement Village 2
Resident
Staff
Number of COVID-19 cases
0
1
2
3
4
5
10141822263034384246501 5 9 1317212529
2020
2021
Epidemiologic week of diagnosis

**Supplementary Figure c.** Number of SARS-CoV-2 cases among LTCFs reporting large outbreaks of SARS-CoV-2 cases, South Africa, 5 March 2020 – 31 July 2021, n=24
